# 

**Published:** 1899-11

**Authors:** 


### CONTENTS.

ORIGINAL COMMUNICATIONS—

A REPORT OF SOME SARCOMATA OF THE JAWS. By W. H. Hudson, M.D., Lafayette, Ala. .......... 577

SOME OF THE DANGERS OF AN IMPURE MEAT AND MILK SUPPLY. By W. H. Dalrymple, M.R.C.V.S. .......... 590

ADVICE TO GONORRHEAL PATIENTS. By W. L. Champion, M.D., Atlanta, Ga. .......... 599

A GENERAL CONSIDERATION OF DISEASES OF EAR, NOSE AND THROAT. By Wm. J. Cox, M.D., Ph.G., Gainesville, Ga. .......... 60[illegible]

SOCIETY REPORTS—

ATLANTA SOCIETY OF MEDICINE .......... 60[illegible]

CORRESPONDENCE—

ANSWERS TO CORRESPONDENTS .......... 61[illegible]

EDITORIAL NOTES AND COMMENTS—

THE EVIL EFFECT UPON WOMEN OF PROLONGED STANDING .......... 613

PHILOLOGIC REFORM .......... 616

THE METRIC SYSTEM .......... 617

MEDICAL DEFENCE UNIONS .......... 619

NEWS AND NOTES .......... 621
CONTENTS CONTINUED .......... x

